# Supplementary material for: Development of a Machine Learning Model of Postoperative Acute Kidney Injury Using Non-Invasive Time-Sensitive Intraoperative Predictors
Source: Bioengineering (Basel). 2023 Aug 5;10(8):932. doi: 10.3390/bioengineering10080932 (PMC10451203; doi:10.3390/bioengineering10080932)
Supplement: Supplementary file 1 [file bioengineering-10-00932-s001.zip › bioengineering-2501524-SI.pdf]

Supplemental Material

Development of a Machine Learning Model of Postoperative Acute Kidney Injury Using Non-Invasive Time-Sensitive Intraoperative Predictors

Siavash Zamirpour, Alan E. Hubbard, Jean Feng, Atul J. Butte, Romain Pirracchio and Andrew Bishara

Table S1. Candidate preoperative and intraoperative variables. *A binary indicator for missingness was also a candidate variable. Time-dependent intraoperative variables were encoded as the number of minutes below and above the 2.5%, 5%, 10%, 25%, 50%, 75%, 90%, 95%, and 97.5% population-level percentiles (see Supplementary Table 2). Bolded terms were included in the POSTOP-AKI model.

Preoperative variables	Intraoperative variables
**Last preoperative sCr within 90 days**	Non-invasive blood pressure
**Minimum preoperative sCr within 90 days**	Systolic, diastolic, and mean
Self-reported gender*	Temperature
Age*	**Pulse oximetry perfusion index**
Height*	**Heart rate**
Weight*	Rate pressure product
Body surface area*	Pulse pressure
Body mass index*	Pulse pressure variation
Temperature*	Pleth variability index
Heart rate*	**Urine output per minute**
ASA class and emergency*	**Total estimated blood loss**
Booking case length*	Total estimated blood loss per minute
Surgery-specific risk score*	Total urine output
Number of past cardiovascular diagnoses	Total urine output per minute
	Total fluids in
	Total fluids in per minute
	Total pressor dose
	Total pressor dose per kilogram
	Total pressor dose per kilogram per minute

Table S2. Distributions of intraoperative variables. To prevent longer cases from being overrepresented in these distributions, 100 values of each variable were resampled with replacement from each case prior to calculating the quantiles. Bolded thresholds were included in the POSTOP-AKI model.

Variable	2.5%	5%	10%	25%	50%	75%	90%	95%	97.5%
Systolic BP (mmHg)	80	85	90	100	112	129	147	160	171
Diastolic BP (mmHg)	45	48	51	55	63	72	82	88	94
Mean BP (mmHg)	59	63	67	73	81	92	104	112	119
Temperature (°F)	93.0	94.3	95.0	96.1	97.2	98.1	99.0	99.6	100.6
Perfusion index (%)	0	0.1	0.3	**0.8**	1.9	3.4	5	6.2	7.2
Heart rate (BPM)	48	51	55	63	73	**85**	97	105	112
Rate pressure product (BPM*mmHg)	4672	5125	5673	6780	8308	10295	12535	14080	15600
Pulse pressure (mmHg)	23	27	32	39	49	62	77	86	95
Pulse pressure variation (%)	0	0	2	4	7	12	20	26	33
Pleth variability index (%)	4	4	6	9	14	21	30	37	43
Urine output (mL/min)	0.00	0.00	0.00	**31.25**	85.71	181.82	323.08	457.14	642.86

Table S3. POSTOP-AKI model coefficients for the outcome of postoperative increase in serum creatinine ([maximum 48-hour postoperative sCr] – [last 90-day preoperative sCr]) in mg/dL. The model intercept is 0.02559.

Terms	Model Coefficient
Last 90-day preoperative sCr per 1 mg/dL	0.05154
Minimum 90-day preoperative sCr per 1 mg/dL	-0.05630
Minutes urine output ≤ 31.29 mL/hr per 1 minute	-0.0001348
Total estimated blood loss per 1 mL	2.771E-05
Minutes pulse oximetry heart rate > 85 per 1 minutes	0.0001395
Minutes pulse oximetry perfusion index ≤ 0.8% per 1 minutes	-7.588E-05
Last 90-day preoperative sCr per 1 mg/dL × Minutes urine output ≤ 31.29 mL/hr per 1 minute	0.03246
Last 90-day preoperative sCr per 1 mg/dL × Total estimated blood loss per 1 mL	-1.162E-05
Last 90-day preoperative sCr per 1 mg/dL × Minutes pulse oximetry heart rate > 85 bpm per 1 minute	-6.738E-05
Minimum 90-day preoperative sCr per 1 mg/dL × Minutes urine output ≤ 31.29 mL/hr per 1 minute	0.0003366
Minutes urine output ≤ 31.29 mL/hr per 1 minute × Minutes pulse oximetry perfusion index ≤ 0.8% per 1 minute	0.0001947
Minimum 90-day preoperative sCr per 1 mg/dL × Minutes pulse oximetry heart rate > 85 bpm per 1 minutes	8.537E-09
Total estimated blood loss per 1 mL × Minutes pulse oximetry heart rate > 85 bpm per 1 minutes	1.238E-08
Total estimated blood loss per 1 mL × Minutes pulse oximetry perfusion index ≤ 0.8% per 1 minutes	-5.017E-08

Table S4. Main term coefficients in the training set for the outcome of postoperative increase in serum creatinine.

Main Terms	Beta	95% CI
Last 90-day preoperative sCr per 0.1 mg/dL	0.011	0.0055 – 0.017
Minimum 90-day preoperative sCr per 0.1 mg/dL	0.0012	-0.0050 – 0.0070
Minutes urine output ≤ 31.29 mL/hr per 30 minutes	0.0063	0.0030 – 0.0097
Total estimated blood loss per 100 mL	0.0014	-0.000223 – 0.0025
Minutes pulse oximetry heart rate > 85 bpm per 30 minutes	0.0036	0.00157 – 0.0060
Minutes pulse oximetry perfusion index ≤ 0.8% per 30 minutes	0.0027	0.00059 – 0.0051

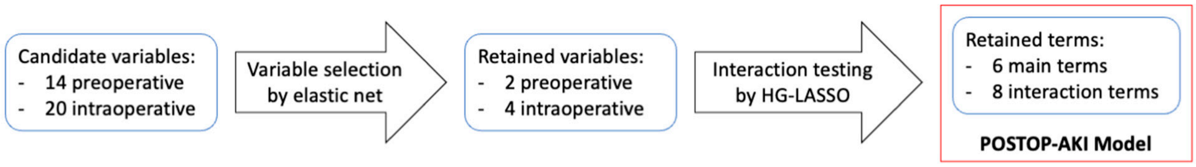

Figure S1. Variable selection and model building process. HG-LASSO, hierarchical group least absolute shrinkage and selection operator; POSTOP-AKI, Perfusion Optimized Score TO Predict AKI

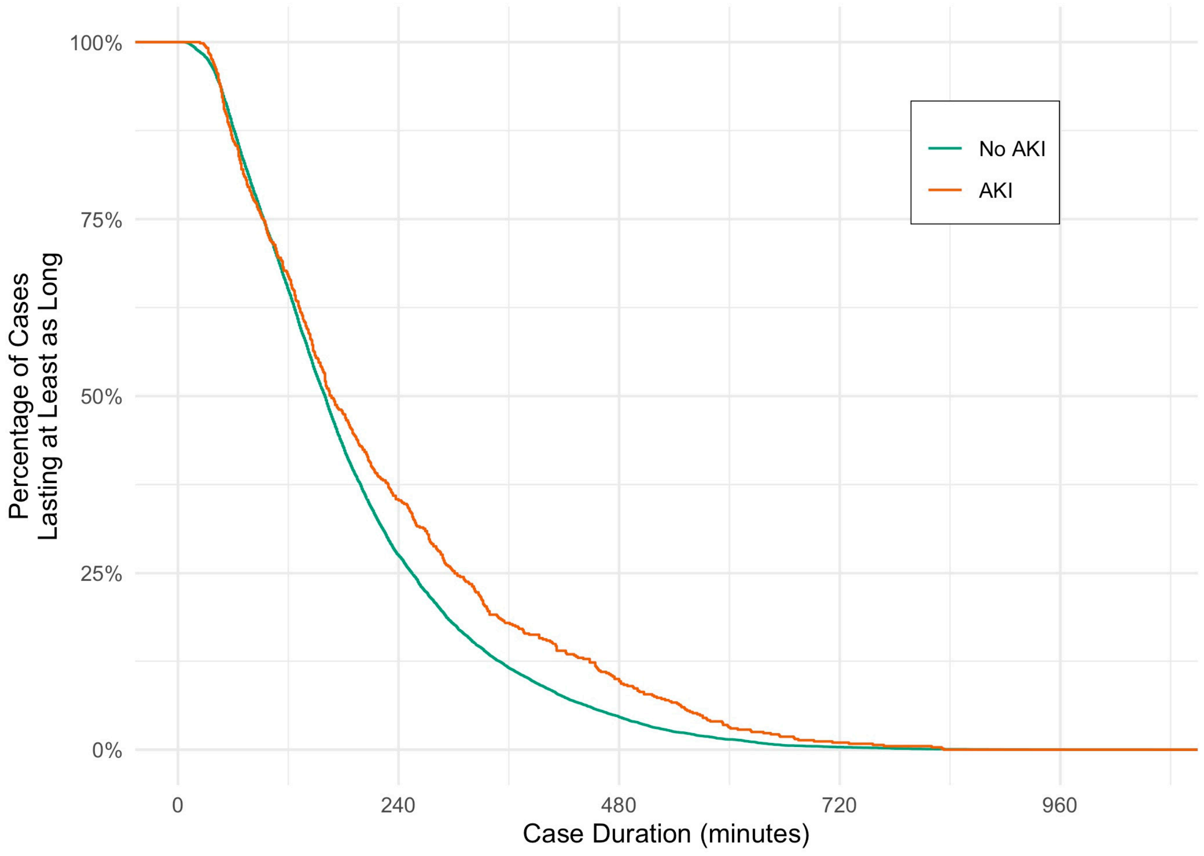

Figure S2. Distribution of case duration.

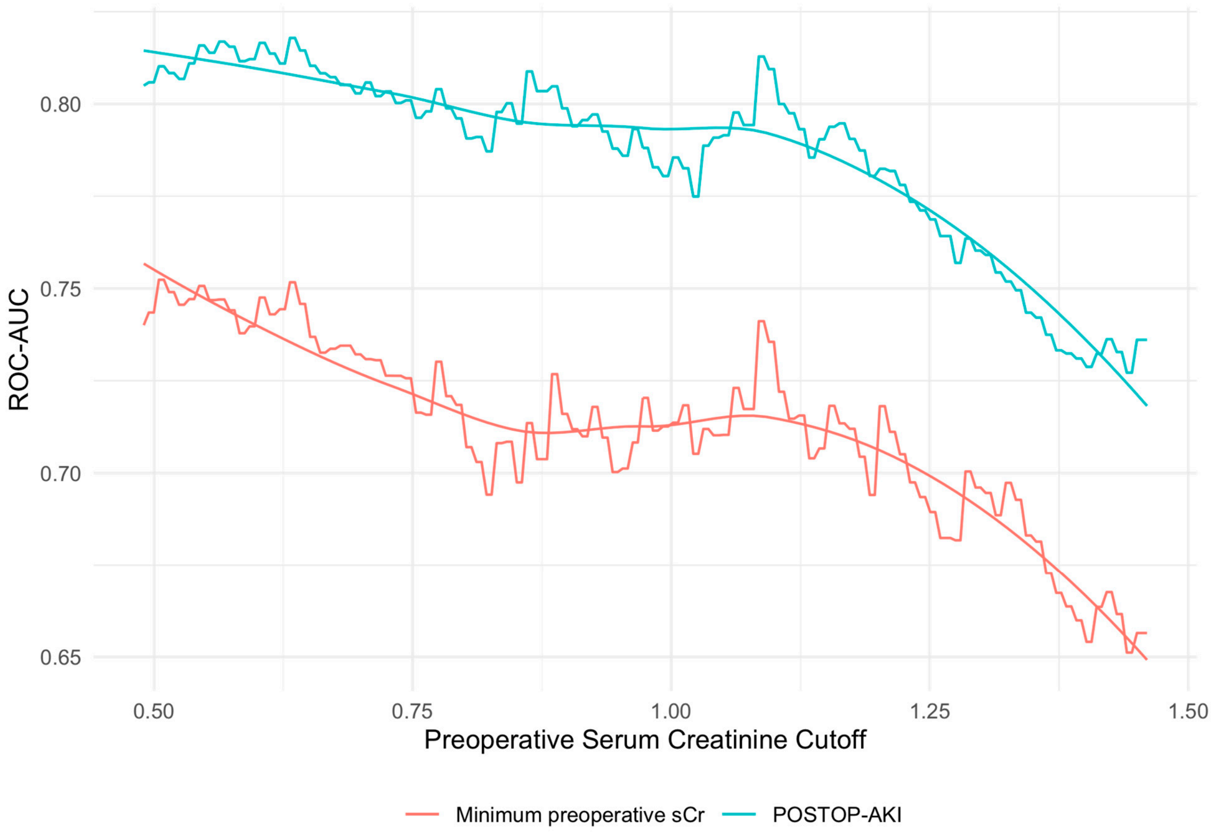

Figure S3. Predictive performance of the POSTOP-AKI model as a function of cutoffs in the 90-day minimum preoperative sCr. For each cutoff shown on the x-axis, the y-axis shows the ROC-AUC for the POSTOP-AKI model and a simpler comparison model among the cohort with 90-day minimum preoperative sCr greater than or equal to the cutoff. Smoothed trends by LOESS regression are also shown. The median [IQR] difference in ROC-AUC across the range of cutoffs shown was 0.074 [0.069–0.081]. sCr, serum creatinine; ROC-AUC, receiver operating characteristic area under the curve; LOESS, locally estimated scatterplot smoothing.

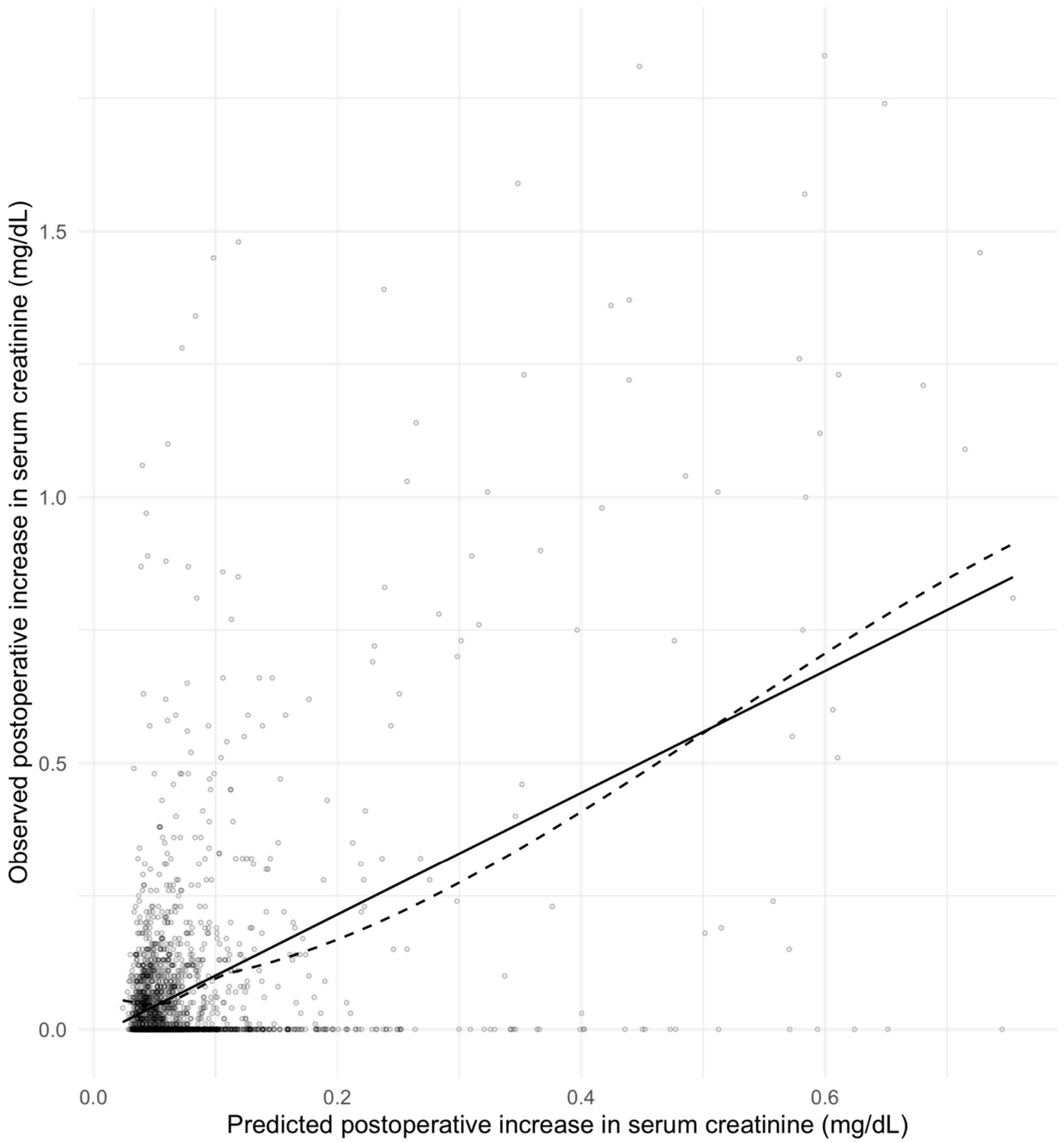

Figure S4. Calibration curve for the POSTOP-AKI model in the validation set. R^2 and RMSE were 0.22 and 0.18, respectively. A linear trend is shown as a solid line and a smoothed trend by LOESS regression is shown as a dashed line. Cases with a postoperative sCr decrease were labeled as having a postoperative sCr increase of 0. One case with an observed postoperative sCr increase of >2 mg/dL is not shown. LOESS, locally estimated scatterplot smoothing.

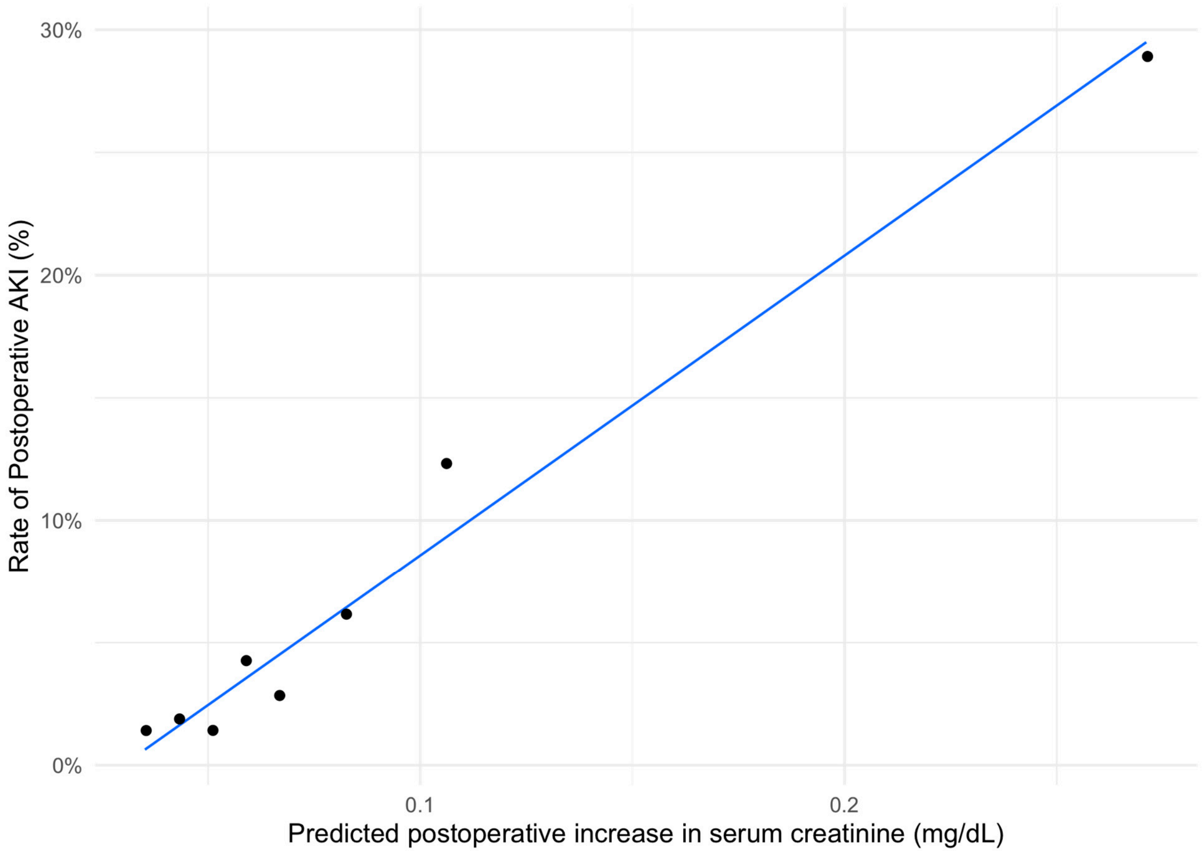

Figure S5. Association between the POSTOP-AKI score and the observed rate of postoperative acute kidney injury in the validation set. Rates of AKI are plotted within deciles of predicted postoperative increase in serum creatinine. AKI, acute kidney injury.

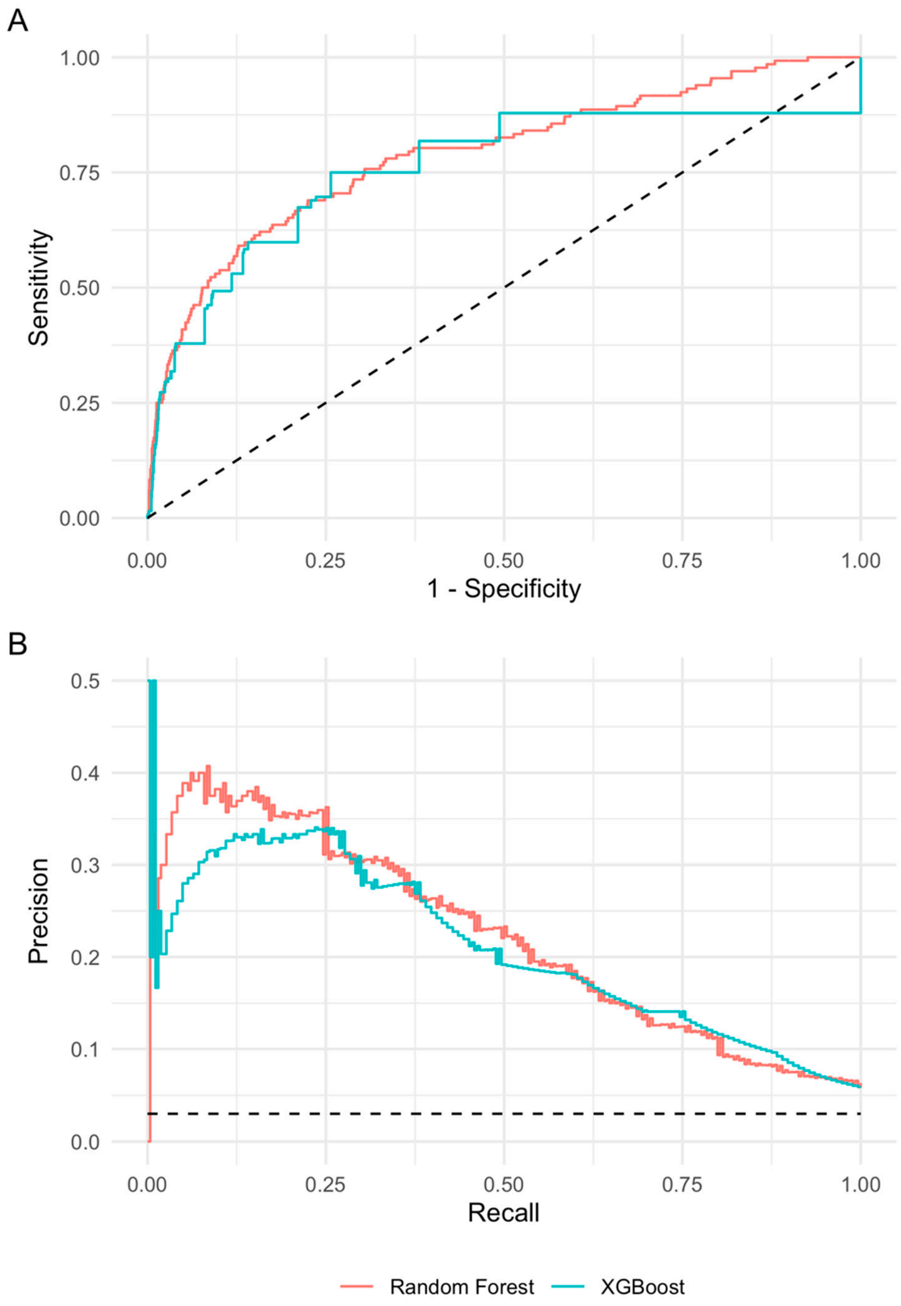

Figure S6. Performance metrics in the validation set for comparison models fit using the variables retained in the elastic net selection step. (**a**) Receiver operating characteristic curves. (**b**) Precision-recall curves.

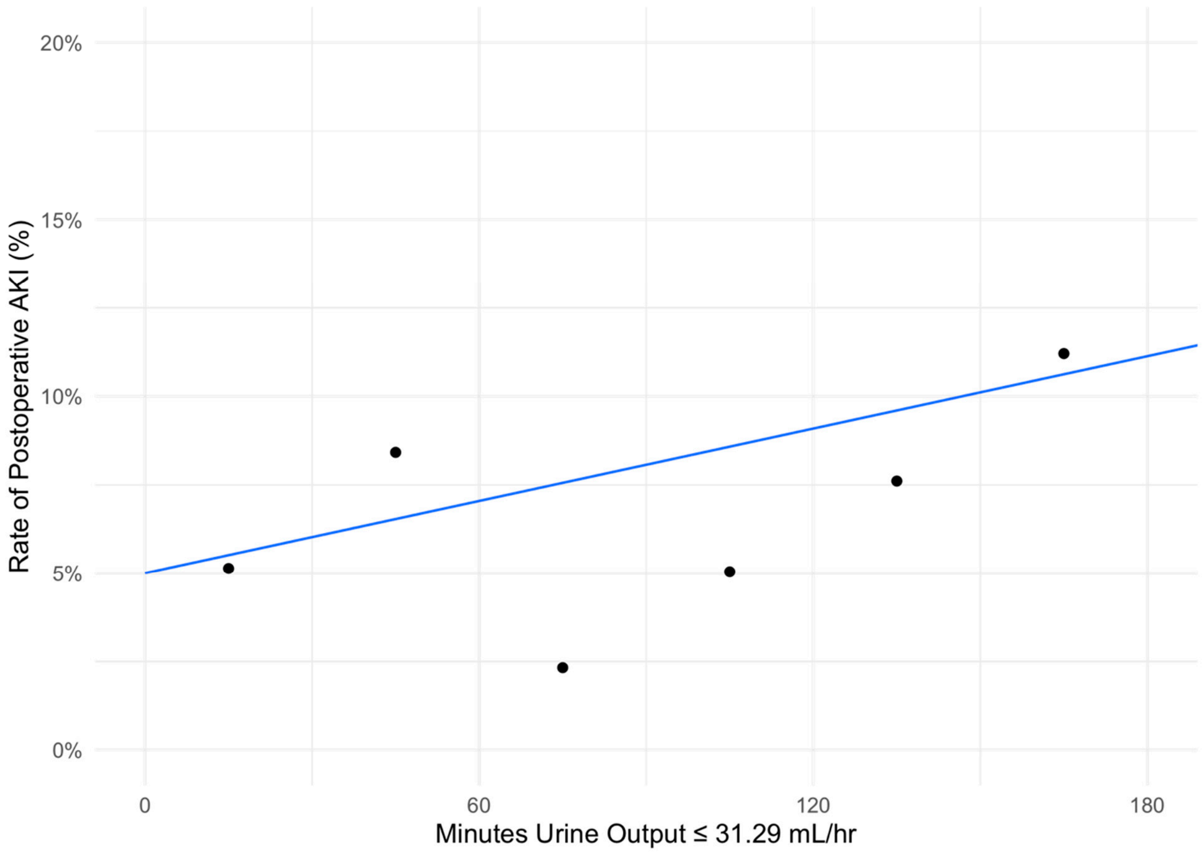

Figure S7. Correlation of duration of meeting intraoperative predictive threshold for urine output and rate of acute kidney injury.

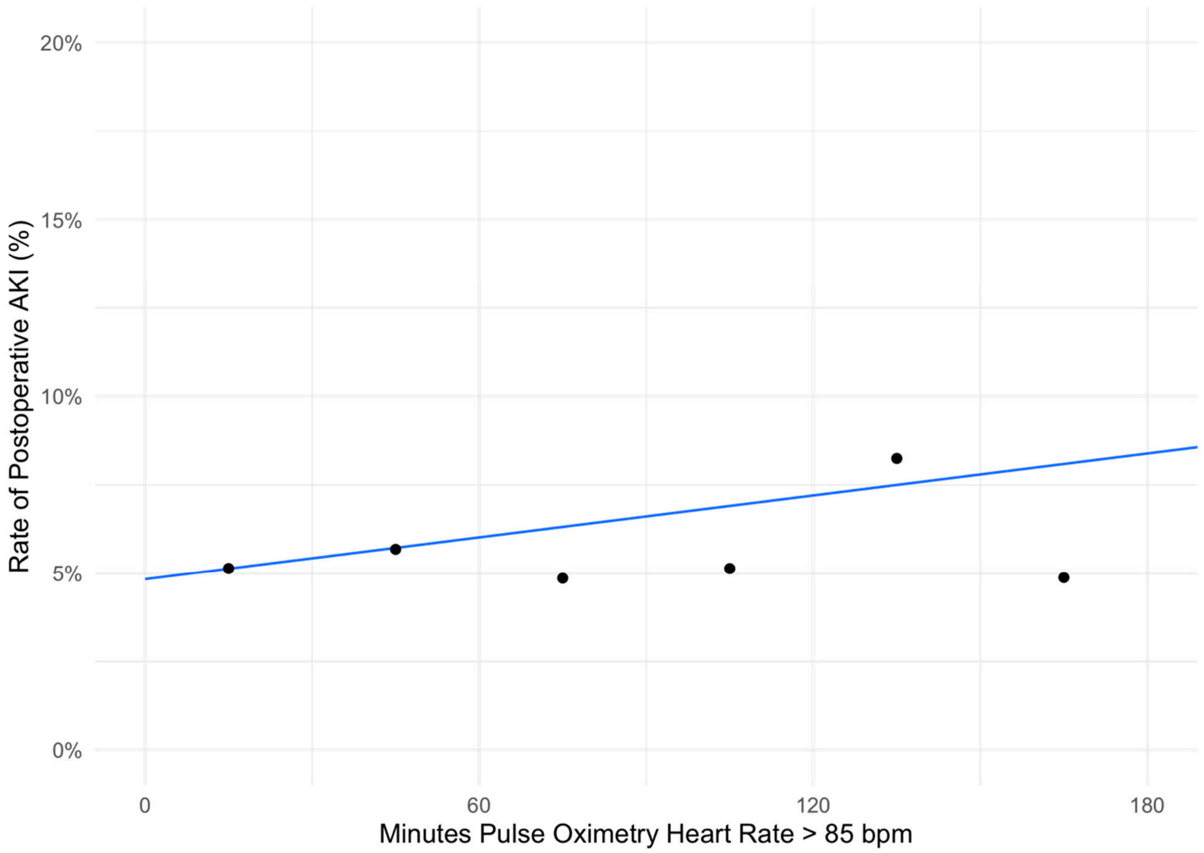

Figure S8. Correlation of duration of meeting intraoperative predictive threshold for pulse oximetry heart rate and rate of acute kidney injury.

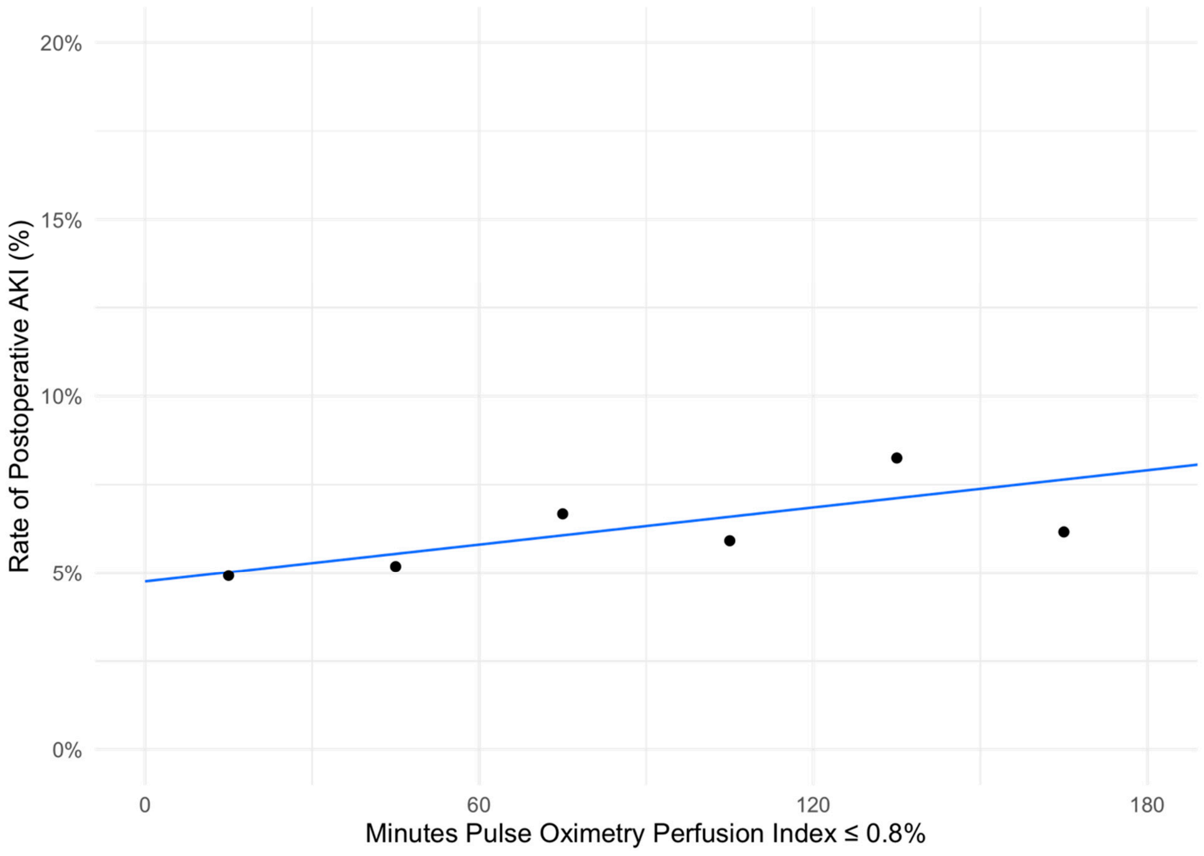

Figure S9. Correlation of duration of meeting intraoperative predictive threshold for pulse oximetry perfusion index and rate of acute kidney injury.

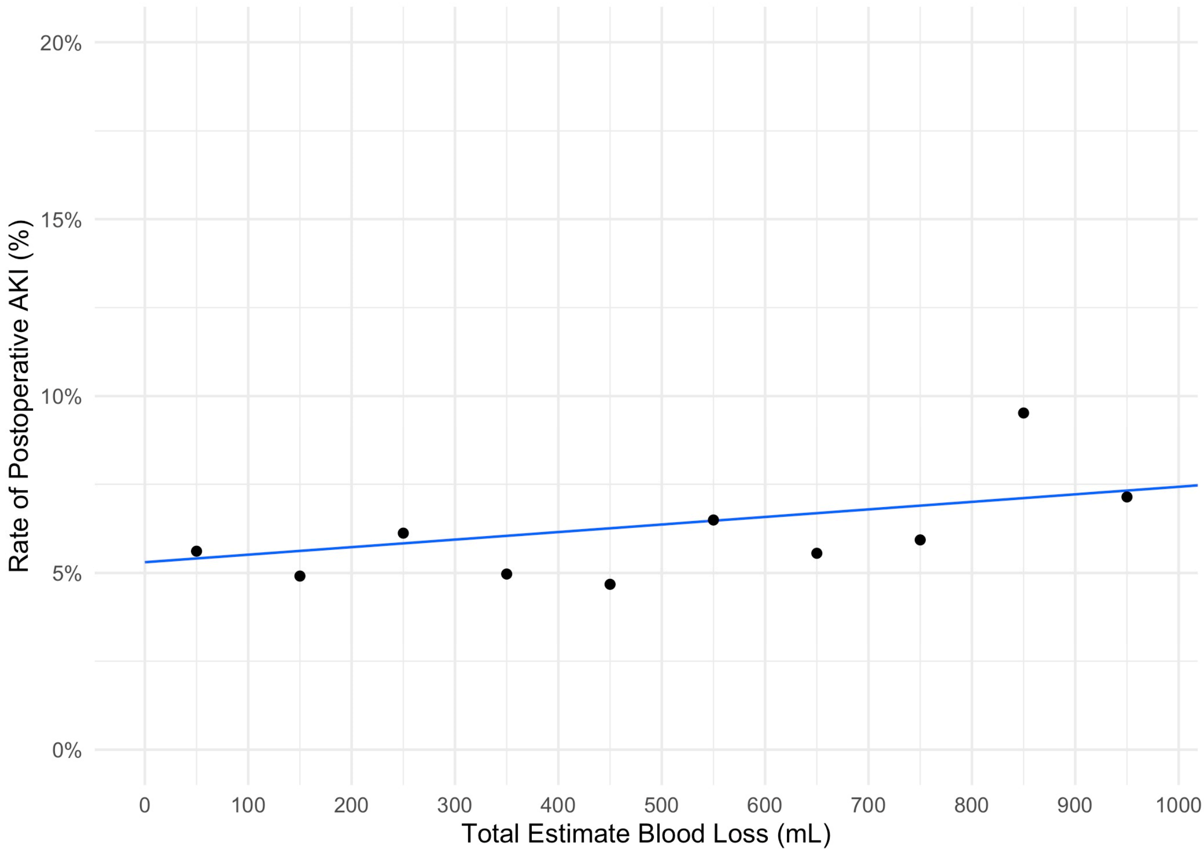

Figure S10. Correlation of total estimated blood loss and rate of acute kidney injury.

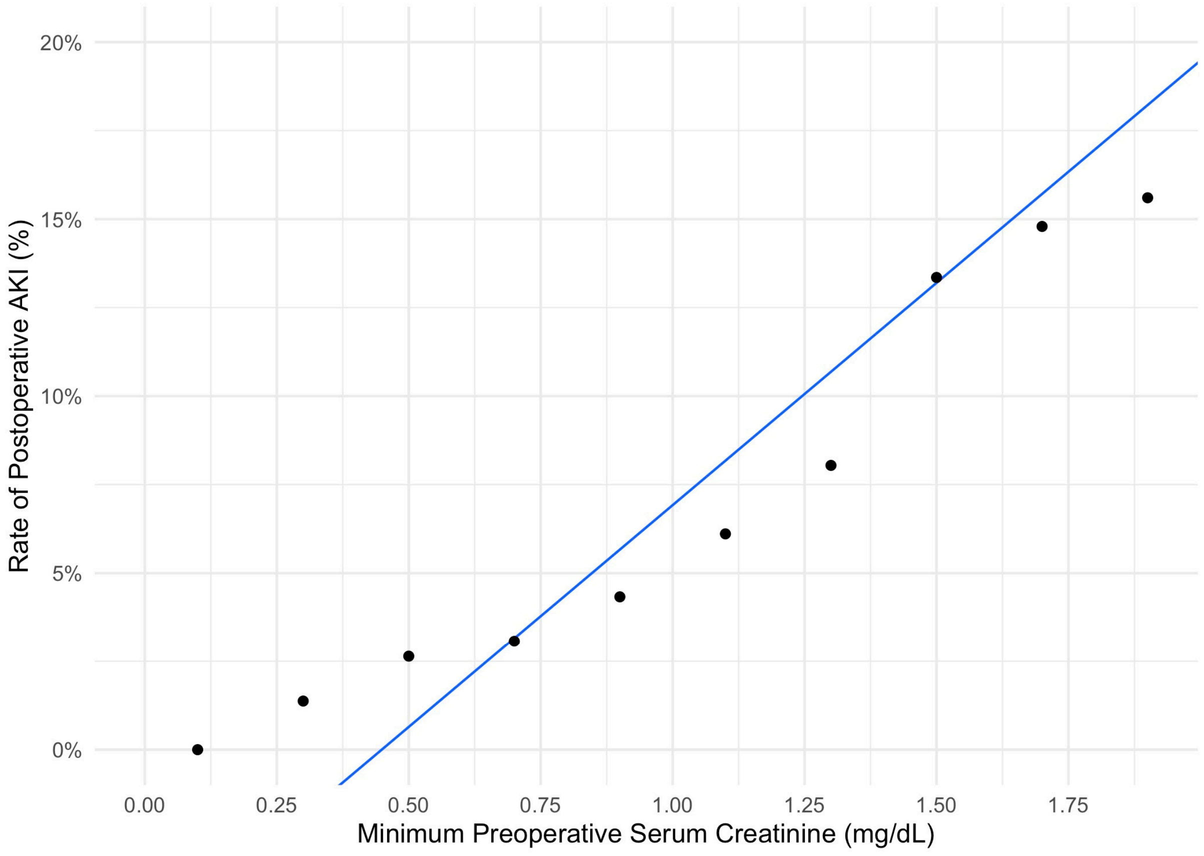

Figure S11. Correlation of 90-day minimum preoperative serum creatinine and rate of acute kidney injury.

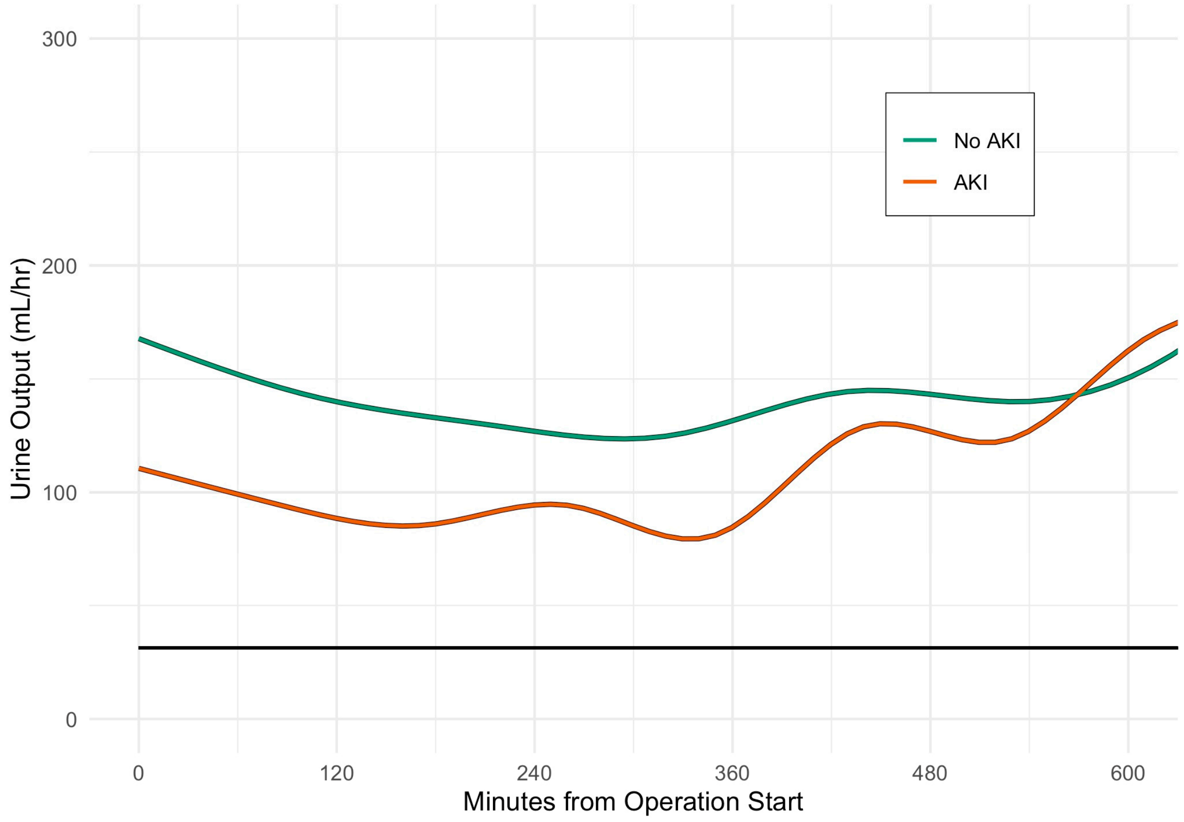

Figure S12. Time-series plot of urine output.

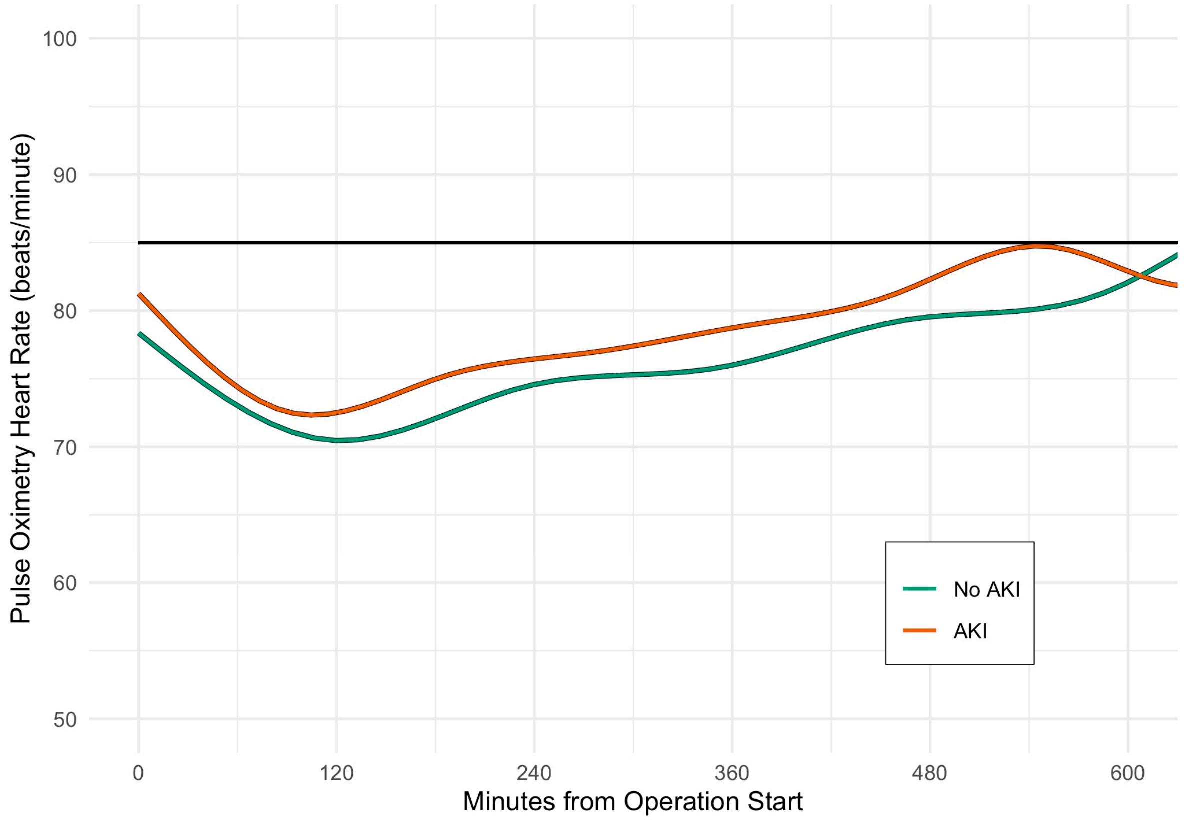

Figure S13. Time-series plot of pulse oximetry heart rate.

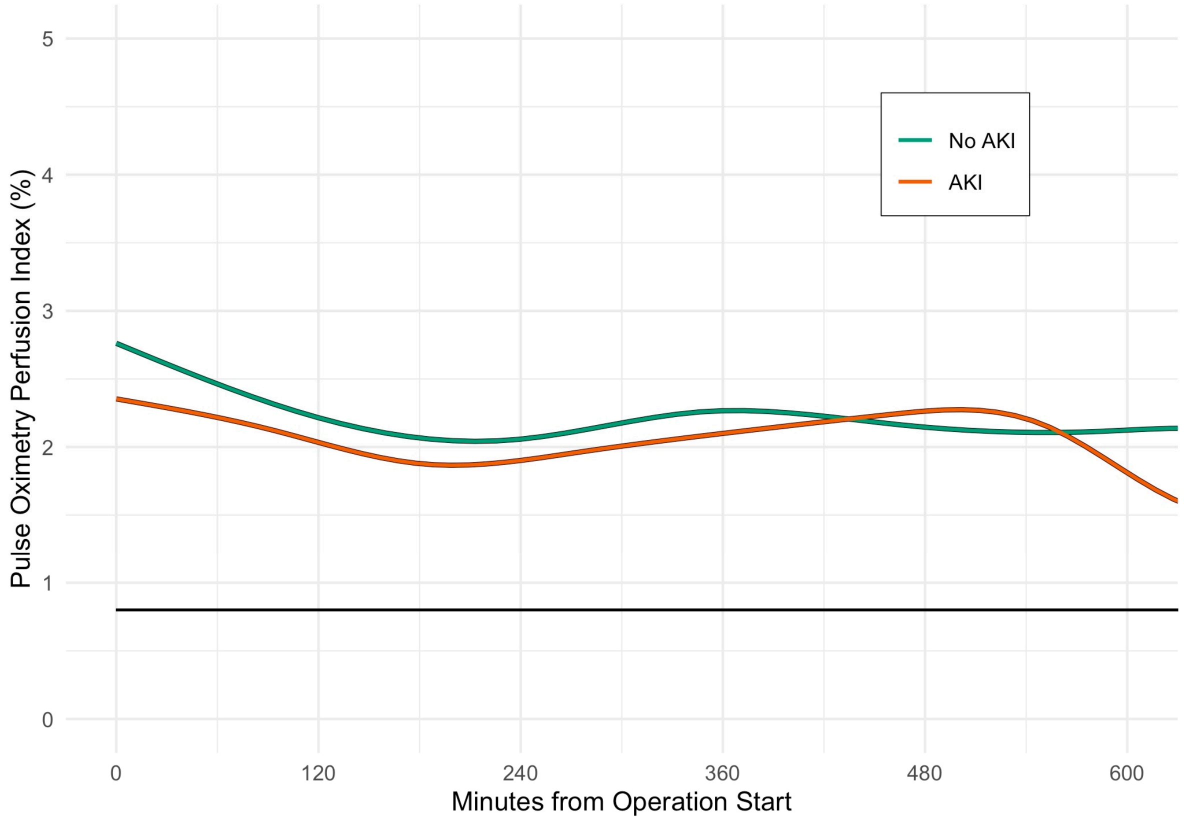

Figure S14. Time-series plot of pulse oximetry perfusion index.

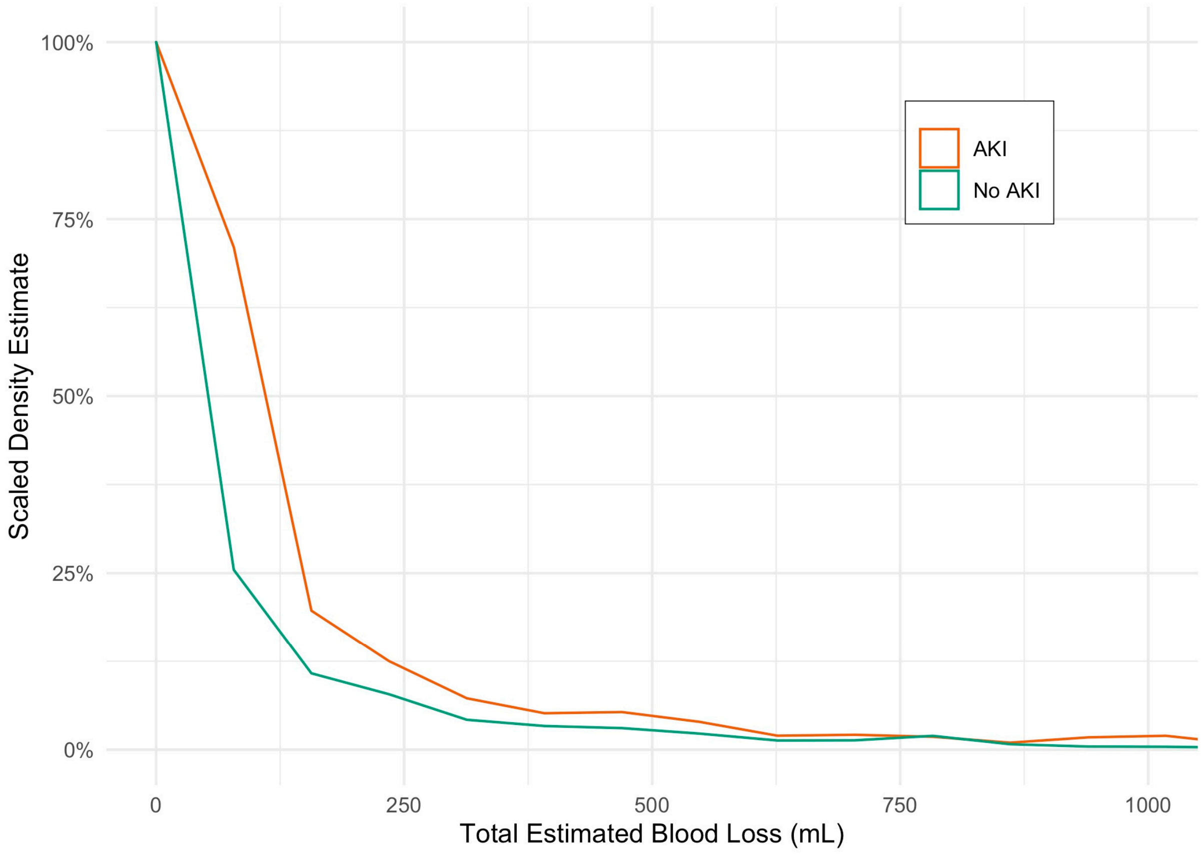

Figure S15. Density plot of total estimated blood loss.

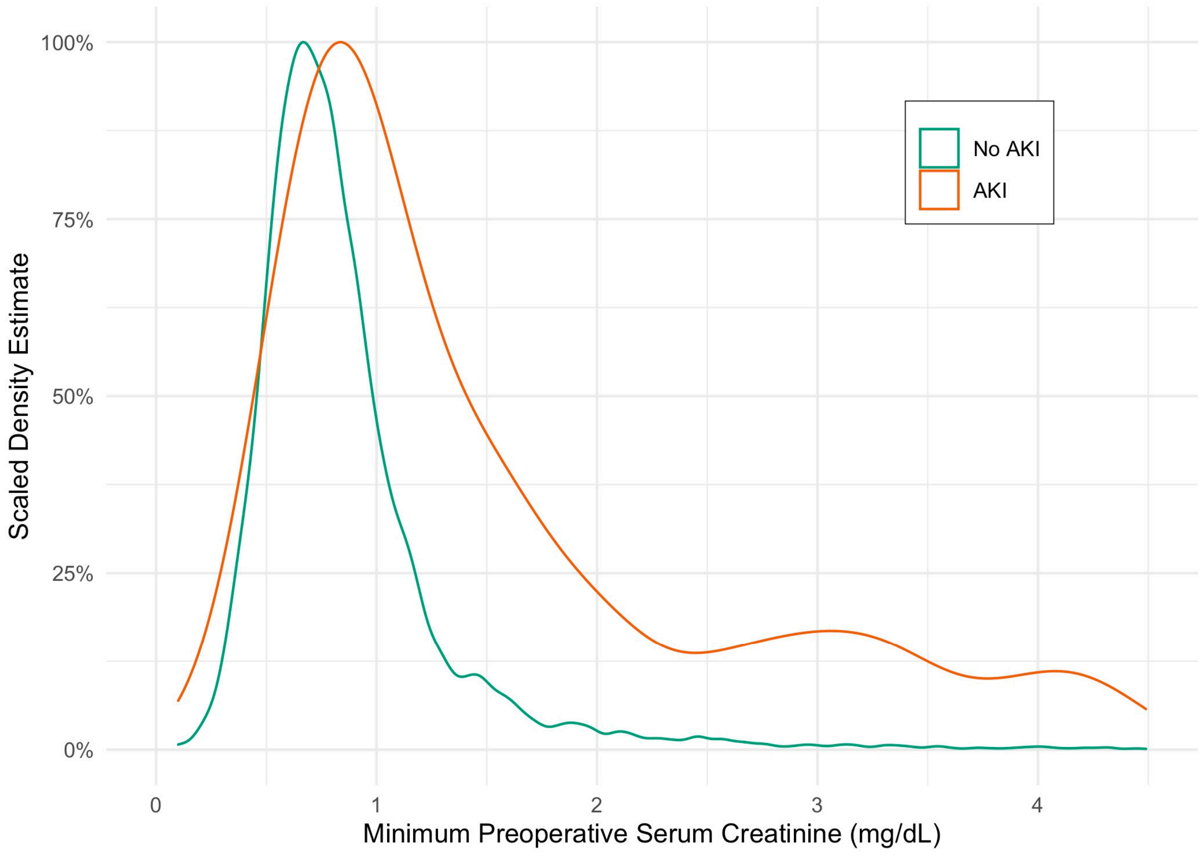

Figure S16. Density plot of 90-day minimum preoperative serum creatinine.

TRIPOD Checklist: Prediction Model Development

Section/Topic	ı	Checklist Item	Page
**Title and abstract**			
Title	1	Identify the study as developing and/or validating a multivariable prediction model, the target population, and the outcome to be predicted.	1
Abstract	2	Provide a summary of objectives, study design, setting, participants, sample size, predictors, outcome, statistical analysis, results, and conclusions.	1
**Introduction**			
Background and objectives	3a	Explain the medical context (including whether diagnostic or prognostic) and rationale for developing or validating the multivariable prediction model, including references to existing models.	1-2
	3b	Specify the objectives, including whether the study describes the development or validation of the model or both.	2
**Methods**			
Source of data	4a	Describe the study design or source of data (e.g., randomized trial, cohort, or registry data), separately for the development and validation data sets, if applicable.	2
	4b	Specify the key study dates, including start of accrual; end of accrual; and, if applicable, end of follow-up.	2
Participants	5a	Specify key elements of the study setting (e.g., primary care, secondary care, general population) including number and location of centres.	2
	5b	Describe eligibility criteria for participants.	2
	5c	Give details of treatments received, if relevant.	5-6
Outcome	6a	Clearly define the outcome that is predicted by the prediction model, including how and when assessed.	3
	6b	Report any actions to blind assessment of the outcome to be predicted.	N/A
Predictors	7a	Clearly define all predictors used in developing or validating the multivariable prediction model, including how and when they were measured.	2-3
	7b	Report any actions to blind assessment of predictors for the outcome and other predictors.	N/A
Sample size	8	Explain how the study size was arrived at.	2
Missing data	9	Describe how missing data were handled (e.g., complete-case analysis, single imputation, multiple imputation) with details of any imputation method.	3
Statistical analysis methods	10a	Describe how predictors were handled in the analyses.	2-4
	10b	Specify type of model, all model-building procedures (including any predictor selection), and method for internal validation.	3
	10d	Specify all measures used to assess model performance and, if relevant, to compare multiple models.	4
Risk groups	11	Provide details on how risk groups were created, if done.	4
**Results**			
Participants	13a	Describe the flow of participants through the study, including the number of participants with and without the outcome and, if applicable, a summary of the follow-up time. A diagram may be helpful.	4-5
	13b	Describe the characteristics of the participants (basic demographics, clinical features, available predictors), including the number of participants with missing data for predictors and outcome.	4-6
Model development	14a	Specify the number of participants and outcome events in each analysis.	4-5
	14b	If done, report the unadjusted association between each candidate predictor and outcome.	6
Model specification	15a	Present the full prediction model to allow predictions for individuals (i.e., all regression coefficients, and model intercept or baseline survival at a given time point).	6
	15b	Explain how to the use the prediction model.	6
Model performance	16	Report performance measures (with CIs) for the prediction model.	6-7
**Discussion**			
Limitations	18	Discuss any limitations of the study (such as nonrepresentative sample, few events per predictor, missing data).	9

Interpretation	19b	Give an overall interpretation of the results, considering objectives, limitations, and results from similar studies, and other relevant evidence.	8-9
Implications	20	Discuss the potential clinical use of the model and implications for future research.	8-9
**Other information**			
Supplementary information	21	Provide information about the availability of supplementary resources, such as study protocol, Web calculator, and data sets.	9-10
Funding	22	Give the source of funding and the role of the funders for the present study.	10

We recommend using the TRIPOD Checklist in conjunction with the TRIPOD Explanation and Elaboration document.

STROBE Statement—checklist of items that should be included in reports of observational studies

	Item No.	Recommendation	Page No.	Relevant text from manuscript
**Title and abstract**	1	(*a*) Indicate the study's design with a commonly used term in the title or the abstract	1	"Development of a Machine Learning Model"<br>"In this retrospective cohort study …"
		(*b*) Provide in the abstract an informative and balanced summary of what was done and what was found	1	"Acute kidney injury (AKI) …" through "Further research is needed to evaluate the model in clinical settings."
**Introduction**				
Background/rationale	2	Explain the scientific background and rationale for the investigation being reported	1-2	"Acute kidney injury (AKI) …" through "indicators of central hypotension."
Objectives	3	State specific objectives, including any prespecified hypotheses	2	"The objective of this study was to characterize non-invasive, time-sensitive in-traoperative predictors of AKI. Our hypothesis was that a limited set of physiologically relevant intraoperative variables provides adequate prediction of postoperative AKI."
**Methods**				
Study design	4	Present key elements of study design early in the paper	2	"We conducted a retrospective cohort study …"
Setting	5	Describe the setting, locations, and relevant dates, including periods of recruitment, exposure, follow-up, and data collection	2	"We conducted a retrospective cohort study from 2016-2022 at the University of California, San Francisco, an urban quaternary academic medical center."
Participants	6	(*a*) *Cohort study*—Give the eligibility criteria, and the sources and methods of selection of participants. Describe methods of follow-up<br>*Case-control study*—Give the eligibility criteria, and the sources and methods of case ascertainment and control selection.	2	"Inclusion criteria included adult operative cases during the study period with ≥ 1 serum creatinine (sCr) value in the 90 days preceding surgery and ≥ 1 serum sCr in the 48 hours following surgery (Figure 1). Exclusion criteria included obstetric, kidney donor and recipient, and arteriovenous fistula cases due to preexisting alteration in renal physiology. For the same reason, we excluded those with last preoperative sCr ≥ 4.5 mg/dL."

		Give the rationale for the choice of cases and controls<br>*Cross-sectional study*—Give the eligibility criteria, and the sources and methods of selection of participants		
		(*b*) *Cohort study*—For matched studies, give matching criteria and number of exposed and unexposed<br>*Case-control study*—For matched studies, give matching criteria and the number of controls per case	N/A	
Variables	7	Clearly define all outcomes, exposures, predictors, potential confounders, and effect modifiers. Give diagnostic criteria, if applicable	2-3	"The main outcome was AKI according to the Kidney Disease Improving Global Outcomes (KDIGO) criteria in the 48 hours following surgery …"<br>"Candidate preoperative and intraoperative predictors were selected based on being routinely measured, noninvasive, or suspected in the literature or the investigators' clinical experience (Table S1)."<br>"We then assessed predictive performance in subpopulations of the validation set defined by cutoffs in the 90-day minimum preoperative sCr due to the influence of preoperative kidney function on the risk of developing postoperative AKI."
Data sources/ measurement	8*	For each variable of interest, give sources of data and details of methods of assessment (measurement). Describe comparability of assessment methods if there is more than one group	2-3	"A surgery-specific risk score was calculated as described previously [22]. Intraoperative variables were recorded at a frequency of 1/60 Hz. Non-invasive blood pressure and urine output were measured intermittently. Non-invasive blood pressure values were linearly interpolated. Urine output was back-calculated as a constant rate to the preceding urine output recording or the start of the case."<br>"Data were abstracted using Opal, an implementation science tool for clinical decision support in anesthesia [24]."
Bias	9	Describe any efforts to address potential sources of bias	2-3	"To prevent longer cases from being overrepresented in these distributions, 100 values of each variable were resampled with replacement from each case prior to calculating the quantiles."<br>"While we attempted to incorporate KDIGO urine output criteria in our definition of postoperative AKI, we found that this was not well-suited to retrospective analysis due to unclear or inconsistent urine output charting practices, as was similarly found in other studies [12]. We restricted

				postoperative follow-up to 48 hours rather than 7 days to avoid misclassifying cases in which AKI was more directly related to features of the postoperative rather than intraoperative course.”
Study size	10	Explain how the study size was arrived at	2	“The study size was not prespecified.”

Quantitative variables	11	Explain how quantitative variables were handled in the analyses. If applicable, describe which groupings were chosen and why	2-3	“Quantiles were determined for each variable at 2.5%, 5%, 10%, 25%, 50%, 75%, 90%, 95%, and 97.5% (Table S2).” “We chose three thresholds for the predicted postoperative increase in sCr to facilitate analyses requiring a binary outcome. The middle threshold of 0.0767 was identified using the Youden index. Because interventions for AKI are relatively low risk, we also demonstrate a lower threshold of 0.05, which has higher sensitivity. 0.05 is also the variance in the postoperative increase in sCr in the training set. We also selected a higher threshold of 0.1, which has a higher positive predictive value, to provide users with scenarios wherein higher risk interventions may be warranted. These thresholds were meant to be demonstrative and should not be interpreted as optimized in external data sets.”
Statistical methods	12	(*a*) Describe all statistical methods, including those used to control for confounding	3-4	“All analyses were carried out in R version 4.1.1. …” through “Trends in retained model variables were further explored by plotting the proportion of non-AKI and AKI cases meeting predictive thresholds for urine output, heart rate, and perfusion index at each minute during the procedure.”
		(*b*) Describe any methods used to examine subgroups and interactions	4	“We then assessed predictive performance in subpopulations of the validation set defined by cutoffs in the 90-day minimum preoperative sCr due to the influence of preoperative kidney function on the risk of developing postoperative AKI. Across 200 equally spaced cutoffs from the 10th percentile (0.49) to the 90th percentile (1.46) of minimum preoperative sCr, we calculated ROC-AUCs in the subpopulation of the val-idation set with minimum preoperative sCr greater than or equal to the cutoff.”
		(*c*) Explain how missing data were addressed	3	“Missing data were imputed as the median for continuous variables and the most common category for categorical variables.”

		(*d*) *Cohort study*—If applicable, explain how loss to follow-up was addressed<br>*Case-control study*—If applicable, explain how matching of cases and controls was addressed<br>*Cross-sectional study*—If applicable, describe analytical methods taking account of sampling strategy	N/A	
		(*e*) Describe any sensitivity analyses	N/A	
**Results**				
Participants	13*	(a) Report numbers of individuals at each stage of study—eg numbers potentially eligible, examined for eligibility, confirmed eligible, included in the study, completing follow-up, and analysed	4-5	"We initially evaluated 77,428 adult operative cases not involving obstetric, kidney transplant, or AV fistula surgery during the study period (Figure 1). 11,212 cases were further evaluated based on existing sCr data in the 90 days preceding and 48 hours following the procedure. 589 cases with preoperative sCr ≥ 4.5 mg/dL were excluded from the analysis, resulting in an analytic set of 10,623 cases. 8519 were randomly assigned to the training set and 2104 to the validation set. There were 469 (5.5%) and 132 (6.3%) cases with AKI in the training and validation sets, respectively."
		(b) Give reasons for non-participation at each stage	4-5	"589 cases with preoperative sCr ≥ 4.5 mg/dL were excluded from the analysis … "
		(c) Consider use of a flow diagram	Figure 1	
Descriptive data	14*	(a) Give characteristics of study participants (eg demographic, clinical, social) and information on exposures and potential confounders	4-6	"Patients had a median [IQR] age of 62 [51, 71] years. 5871 (55.3%) patients were male and 6401 (60.3%) identified as White or Caucasian. 2675 (25.2%) were classified as ASA emergency. The most common operative services were orthopedic surgery (3778, 35.6%), neurological surgery (1350, 12.7%), and general surgery (1611, 15.2%). Median [IQR] booking and actual case durations were 210 [133, 242] minutes and 160 [93, 257] minutes, respectively. Clinical characteristics were well-balanced between the training and vali-dation sets, with SMD < 0.1 for all variables (Table 1), including differences in case du-ration (Figure S2)."

		(b) Indicate number of participants with missing data for each variable of interest	5	"Data missingness (%) was 0 with the exception of 3.2, 2.8, and 4.7 for height, weight, and body mass index; 39 and 37 for preoperative temperature and heart rate; 37 and 36 for ASA class and emergency; 18 and 17 for booking case length and surgical risk score."
		(c) *Cohort study*—Summarise follow-up time (eg, average and total amount)	N/A	
Outcome data	15*	*Cohort study*—Report numbers of outcome events or summary measures over time	4-5	"8519 were randomly assigned to the training set and 2104 to the validation set. There were 469 (5.5%) and 132 (6.3%) cases with AKI in the training and validation sets, respectively."
		Case-control study—Report numbers in each exposure category, or summary measures of exposure	N/A	
		Cross-sectional study—Report numbers of outcome events or summary measures	N/A	
Main results	16	(*a*) Give unadjusted estimates and, if applicable, confounder-adjusted estimates and their precision (eg, 95% confidence interval). Make clear which confounders were adjusted for and why they were included	6-7	"Coefficients of the POSTOP-AKI model are presented in Table S3." "To aid model interpretation, an OLS linear regression model was fit on the training set using the main terms retained in the POSTOP-AKI model (Table S4). Variables retained in the POSTOP-AKI model demonstrated largely linear relationships with the observed rate of AKI, supporting the use of a linear model (Figures S7-11)."
		(*b*) Report category boundaries when continuous variables were categorized	7	"Low, middle, and high score thresholds were 0.05, 0.0767, and 0.1 for the POSTOP-AKI model, respectively, and 0.75, 0.945, and 1.25 for the 90-day minimum preoperative sCr, respectively (Table 2)."
		(*c*) If relevant, consider translating estimates of relative risk into absolute risk for a meaningful time period	N/A	

Other analyses	17	Report other analyses done—eg analyses of subgroups and interactions, and sensitivity analyses	6	"The increased predictive performance of the POSTOP-AKI model was consistent across a range of cutoffs in the 90-day minimum preoperative sCr (Figure S3)."
**Discussion**				
Key results	18	Summarise key results with reference to study objectives	6-7	"Anesthesia is an ideal discipline …" through "more interpretable linear model."
Limitations	19	Discuss limitations of the study, taking into account sources of potential bias or imprecision. Discuss both direction and magnitude of any potential bias	9	"This study has some limitations. …" through "Another important limitation of our study is the possibility that the accuracy of the perfusion index varies in different skin tones, as observed for the pulse oximetry oxygen saturation [41]."
Interpretation	20	Give a cautious overall interpretation of results considering objectives, limitations, multiplicity of analyses, results from similar studies, and other relevant evidence	8-9	"In the POSTOP-AKI model …" through "pulse oximetry oxygen saturation [41]."
Generalisability	21	Discuss the generalisability (external validity) of the study results	8-9	"Our model was developed and validated at a single center, and broader application necessitates validation of the model with external data. Importantly, score thresholds for the binary outcome were intended to be demonstrative and are not optimized for external data. Nevertheless, our data represented a broad range of operative services, anesthesiologists, and surgeons, and the retained model variables have clear physiologic reasons to be predictors of AKI."
**Other information**				
Funding	22	Give the source of funding and the role of the funders for the present study and, if applicable, for the original study on which the present article is based	10	"This research was funded by the Foundation for Anesthesia Education and Research Mentored Research Training Grant (FAER MRTG) to Andrew Bishara."

*Give information separately for cases and controls in case-control studies and, if applicable, for exposed and unexposed groups in cohort and cross-sectional studies.

Note: An Explanation and Elaboration article discusses each checklist item and gives methodological background and published examples of transparent reporting. The STROBE checklist is best used in conjunction with this article (freely available on the Web sites of PLoS Medicine at http://www.plosmedicine.org/, Annals of Internal Medicine at http://www.annals.org/, and Epidemiology at http://www.epidem.com/). Information on the STROBE Initiative is available at www.strobe-statement.org.
